# Online Health Search Via Multidimensional Information Quality Assessment Based on Deep Language Models: Algorithm Development and Validation

**DOI:** 10.2196/42630

**Published:** 2024-05-02

**Authors:** Boya Zhang, Nona Naderi, Rahul Mishra, Douglas Teodoro

**Affiliations:** 1 Department of Radiology and Medical Informatics University of Geneva Geneva Switzerland; 2 Department of Computer Science Université Paris-Saclay Centre national de la recherche scientifique, Laboratoire Interdisciplinaire des Sciences du Numérique Orsay France

**Keywords:** health misinformation, information retrieval, deep learning, language model, transfer learning, infodemic

## Abstract

**Background:**

Widespread misinformation in web resources can lead to serious implications for individuals seeking health advice. Despite that, information retrieval models are often focused only on the query-document relevance dimension to rank results.

**Objective:**

We investigate a multidimensional information quality retrieval model based on deep learning to enhance the effectiveness of online health care information search results.

**Methods:**

In this study, we simulated online health information search scenarios with a topic set of 32 different health-related inquiries and a corpus containing 1 billion web documents from the April 2019 snapshot of Common Crawl. Using state-of-the-art pretrained language models, we assessed the quality of the retrieved documents according to their usefulness, supportiveness, and credibility dimensions for a given search query on 6030 human-annotated, query-document pairs. We evaluated this approach using transfer learning and more specific domain adaptation techniques.

**Results:**

In the transfer learning setting, the usefulness model provided the largest distinction between help- and harm-compatible documents, with a difference of +5.6%, leading to a majority of helpful documents in the top 10 retrieved. The supportiveness model achieved the best harm compatibility (+2.4%), while the combination of usefulness, supportiveness, and credibility models achieved the largest distinction between help- and harm-compatibility on helpful topics (+16.9%). In the domain adaptation setting, the linear combination of different models showed robust performance, with help-harm compatibility above +4.4% for all dimensions and going as high as +6.8%.

**Conclusions:**

These results suggest that integrating automatic ranking models created for specific information quality dimensions can increase the effectiveness of health-related information retrieval. Thus, our approach could be used to enhance searches made by individuals seeking online health information.

## Introduction

In today’s digital age, individuals with diverse information needs, medical knowledge, and linguistic skills [1] turn to the web for health advice and to make treatment decisions [2]. The mixture of facts and rumors in online resources [3] makes it challenging for users to discern accurate content [4]. To provide high-quality resources and enable properly informed decision-making [5], information retrieval systems should differentiate between accurate and misinforming content [6]. Nevertheless, search engines rank documents mainly by their relevance to the search query [7], neglecting several health information quality concerns. Moreover, despite attempts by some search engines to combat misinformation [8], they lack transparency in terms of the methodology used and performance evaluation.

*Health misinformation* is defined as health-related information that is inaccurate or misleading based on current scientific evidence [9,10]. Due to the lack of health literacy for nonprofessionals [11] and the rise of the infodemic phenomenon [12]—the rapid spread of both accurate and inaccurate information about a medical topic on the internet [13]—health misinformation has become increasingly prevalent online. Topics related to misinformation, such as “vaccine” or “the relationship between coronavirus and 5G” have gained scientific interest across social media platforms like Twitter and Instagram [14-16] and among various countries [17]. Thus, the development of new credibility-centered search methods and assessment measures is crucial to address the pressing challenges in health-related information retrieval [18].

In recent years, numerous approaches have been introduced in the literature to categorize and assess misinformation according to multiple dimensions. Hesse et al [19] proposed 7 dimensions of *truthfulness*, which include *correctness, neutrality, comprehensibility, precision, completeness, speaker trustworthiness*, and *informativeness*. On the other hand, van der Linden [20] categorized an infodemic into 3 key dimensions: *susceptibility*, *spread*, and *immunization*. Information retrieval shared tasks, such as the Text Retrieval Conference (TREC) and the Conference and Labs of the Evaluation Forum (CLEF), have also started evaluating quality-based systems for health corpora using multiple dimensions [21,22]. The CLEF eHealth Lab Series proposed a benchmark to evaluate models according to the *relevance*, *readability*, and *credibility* of the retrieved information [23]. The TREC Health Misinformation Track 2021 proposed further metrics of *usefulness*, *supportiveness*, and *credibility* [24]. These dimensions also appear in the TREC Health Misinformation Track 2019 as *relevancy*, *efficacy*, and *credibility*, respectively. Additionally, models by Solainayagi and Ponnusamy [25] and Li et al [26] incorporated similar dimensions, emphasizing source *reliability* and the *credibility* of statements. These metrics represent some of the initial efforts to quantitatively assess the effectiveness of information retrieval engines in sourcing high-quality information, marking a shift from the traditional query-document relevance paradigm [27,28]. Despite their variations, these information quality metrics focus on the following 3 main common topics: (1) *relevancy* (also called *usefulness* or *informativeness*) of the source to the search topic, (2) *correctness* (also called *supportiveness* or *efficacy*) of the information according to the search topic, and (3) *credibility* (also called *trustworthiness*) of the source.

Thanks to these open shared tasks, several significant methodologies have been developed to improve the search for higher-quality health information. Although classical bag-of-words–based methods outperform neural network approaches in detecting health-related misinformation when training data are limited [29], more advanced approaches are needed for web content. Specifically, research has proven the effectiveness of a hybrid approach that integrates classical handcrafted features with deep learning [18]. Further to this, multistage ranking systems [30,31], which couple the system with a label prediction model or use T5 [32] to rerank Okapi Best Match 25 (BM25) results, have been proposed. Particularly, Lima et al [30] considered the stance of the search query and engaged 2 assessors for an interactive search, integrating a continuous active learning method [33]. This approach sets a baseline of human effort in separating helpful from harmful web content. Despite their success, these models often do not take into account the different information quality aspects in their design.

In this study, we aimed to investigate the impact of multidimensional ranking on improving the quality of retrieved health-related information. Due to its coverage of the main information quality dimensions used in the scientific literature, we followed the empirical approach proposed in the TREC 2021 challenge, which considers *usefulness*, *supportiveness*, and *credibility* metrics, to propose a multidimensional ranking model. Using deep learning–based pretrained language models [34] through transfer learning and domain adaption approaches, we categorized the retrieved web resources according to different information quality dimensions. Specialized quality-oriented ranks obtained by reranking components were then fused [32] to provide the final ranked list. In contrast to prior studies, our approach relied on the automatic detection of harmful (or inaccurate) claims and used a multidimensional information quality model to boost helpful resources.

The main contributions of this work are 3-fold. We propose a multidimensional ranking model based on transfer learning and showed that it achieves state-of-the-art in automatic (ie, when the query stance is not provided) quality-centered ranking evaluations. We investigated our approach in 2 learning settings—transfer learning (ie, without query relevance judgments) and domain adaptation (ie, with query relevance judgments from a different corpus)—and demonstrated that they are capable of identifying more helpful documents than harmful ones, obtaining +5% and +7% help and harm compatibility scores, respectively. Last, we investigated how the combination of models specialized in different information dimensions impacts the quality of the results, and our analysis suggests that multidimensional aspects are crucial for extracting high-quality information, especially for unhelpful topics.

## Methods

In this section, we introduce our search model based on multidimensional information quality aspects. We first describe the evaluation benchmark. We then detail the implementation methodology and describe our evaluation experiments using transfer learning and domain adaptation strategies.

### TREC Health Misinformation Track 2021 Benchmark

#### Benchmark Data Set

To evaluate our approach, we used the TREC Health Misinformation Track 2021 benchmark [35] organized by the National Institute of Standards and Technology (NIST) [36]. The TREC Health Misinformation Track 2021 benchmark simulates web searches for specific health issues and interventions against a collection of English web documents [37]. For each topic, the benchmark annotates the quality of the retrieved web documents using a pooling approach, in which the top retrieved documents by systems participating in the challenge are evaluated according to their usefulness, correctness, and credibility and subsequently labeled as helpful or harmful. In this context, helpful documents are defined as those supportive of helpful treatments or that try to dissuade the reader from using unhelpful treatments, while harmful documents encourage the use of unhelpful treatments or dissuade the reader from using helpful treatments [24]. See Table S1 in Multimedia Appendix 1 for more detail on the annotation.

#### Health-Related Topics

A topic in the TREC Health Misinformation Track 2021 benchmark consists of a health issue, an intervention, a query that connects the corresponding intervention to the health problem, and a description that resembles the web search question using natural language. NIST only provided assessments for 35 of the initial 50 topics. Among the assessed topics, 3 were further excluded due to the absence of harmful documents. Consequently, the benchmark consisted of 32 topics: 14 labeled as helpful and 18 labeled as unhelpful. For these queries, a total of 6030 query-document pairs were human-annotated according to different scales of usefulness, correctness, and credibility scores. A “helpful topic” refers to an intervention beneficial for treating a health issue, while an “unhelpful topic” indicates an ineffective intervention. The stance is supported by evidence from a credible source. Table 1 presents examples of the queries and descriptions of helpful and unhelpful topics.

**Table 1:** Examples of helpful and unhelpful topics with query and description.

Number	Query	Description	Stance
106	vitamin b12 sun exposure vitiligo	Can vitamin b12 and sun exposure together help treat vitiligo?	Helpful
102	tepid sponge bath reduce fever children	Is a tepid sponge bath a good way to reduce fever in children?	Unhelpful

#### Web Corpus

We used the Colossal Clean Crawled Corpus (C4), a collection of English-language web documents sourced from the public Common Crawl web scrape [38]. The corpus comprises 1 billion English documents from the April 2019 snapshot. To illustrate the contradictory nature of the web information within the corpus, in Table 2, we present 2 documents relevant to topic 102: “tepid sponge bath reduce fever in children.” Although an article advises against the intervention (“Do Not Use Sponging to Reduce a Fever”), another article advises it could be a viable option (“Sponging is an option for high fevers”).

**Table 2:** Examples of useful but contradictory documents for Topic 102: “Is a tepid sponge bath a good way to reduce fever in children?”.

Article information	Article 1	Article 2
Doc ID	en.noclean.c4-train.07165-of-07168.96468	en.noclean.c4-train.00001-of-07168.126948
Time stamp	2019-04-25T18:00:17Z	2019-04-23T20:13:31Z
Text	[...] Do Not Use Sponging to Reduce a Fever. It is not recommended that you use sponging to reduce your child’s fever. There is no information that shows that sponging or tepid baths improve your child’s discomfort associated with a fever or an illness. Cool or cold water can cause shivering and increase your child’s temperature. Also, never add rubbing alcohol to the water. Rubbing alcohol can be absorbed into the skin or inhaled, causing serious problems such as a coma. [...]	[...] Sponging With Lukewarm Water: Note: Sponging is an option for high fevers, but not required. It is rarely needed. When to Use: Fever above 104° F (40° C) AND doesn’t come down with fever meds. Always give the fever medicine at least an hour to work before sponging. How to Sponge: Use lukewarm water (85 - 90° F) (29.4 - 32.2° C). Sponge for 20-30 minutes. If your child shivers or becomes cold, stop sponging. [...]
URL	https://patiented.solutions.aap.org/	https://childrensclinicofraceland.com/

### Quality-Based Multidimensional Ranking Conceptual Model

#### Phases

**Figure 1:**
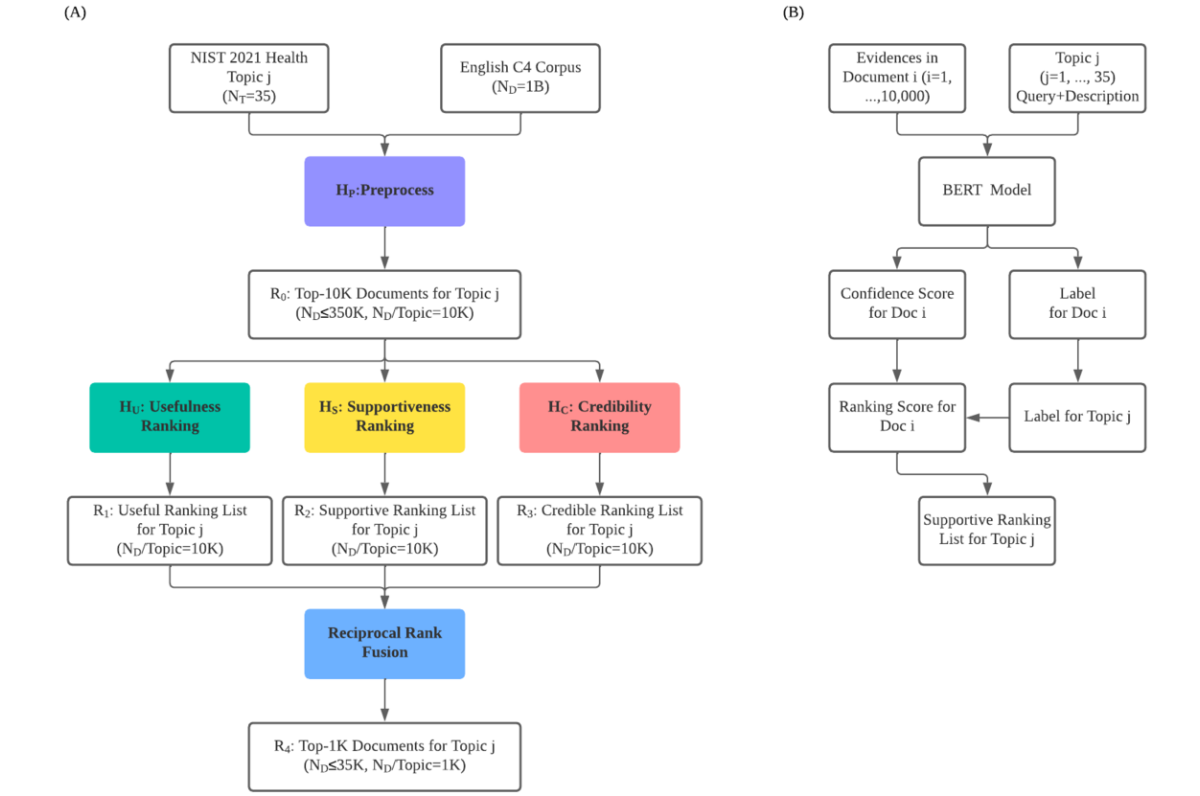
Quality-based multidimensional ranking models: (A) general pipeline, (B) supportiveness model for the transfer learning approach. BERT: Bidirectional Encoder Representations from Transformers; C4: Colossal Clean Crawled Corpus; NIST: National Institute of Standards and Technology.

The quality-based multidimensional ranking model proposed in this work is presented in Figure 1A. The information retrieval process can be divided into 2 phases: *preprocessing* and *multidimensional ranking*. In the preprocessing phase, for a given topic *j*, *N_D_* documents were retrieved based on their relevance (eg, using a BM25 model) [39]. In the multidimensional ranking phase, we further estimated the quality of the retrieved subset of documents according to the usefulness, supportiveness, and credibility dimensions. In the following sections, we describe the multidimensional ranking approach and its implementation using transfer learning and domain adaption. We then describe the preprocessing step, which can be performed based on sparse or dense retrieval engines.

#### Multidimensional Ranking

To provide higher-quality documents at the top ranks, we proposed using a set of machine learning models trained to classify documents according to the usefulness, supportiveness, and credibility dimensions. For the initial rank list obtained in the preprocessing phase (see details in the following sections), the documents were reranked in parallel according to the following strategies for usefulness, supportiveness, and credibility.

##### Usefulness

The usefulness dimension is defined as *the extent to which the document contains information that a search user would find useful in answering the topic’s question*. In this sense, it defines how pertinent a document is to a given topic. Thus, to compute the usefulness of retrieved documents, topic-document similarity models based on pretrained language models, such as Bidirectional Encoder Representations from Transformers (BERT)–base [40], mono-BERT-large [41], and ELECTRA [42], could be used. Given a topic-document pair, the language model infers a score that gives the level of similarity between the 2 input text passages. Although bag-of-words models, such as BM25, provide a strong baseline for usefulness, they do not consider word relations by learning context-sensitive representations as is the case with the pretrained language models, which are used to enhance the quality of the original ranking [28].

##### Supportiveness

The supportiveness dimension defines whether *the document supports or dissuades the use of the treatment in the topic’s question*. Therefore, it defines the stance of the document on the health topic. In this dimension, documents are identified under 3 levels: (1) supportive (ie, the document supports the treatment), (2) dissuasive (ie, the document refutes the treatment), and (3) neutral (ie, the document does not contain enough information to make the decision) [35]. To compute the supportiveness of a document to a given query, the system should be optimized so that documents that are either supportive, if the topic is helpful, or dissuasive, if the topic is unhelpful, are boosted to the top of the ranking list, which means that correct documents are boosted and misinforming documents are downgraded.

##### Credibility

The credibility dimension defines *whether the document is considered credible by the assessor*, that is, how trustworthy the source document is. To compute this dimension, the content of the document itself could be used (eg, leveraging language features, such as readability [43]), which is assessable using the Simple Measure of Gobbledygook index [44]. Moreover, document metadata could be also used, such as incoming and outcoming links, which can be calculated with link analysis algorithms [45], and URL addresses considered to be trusted sources [46].

#### Transfer Learning Implementation

To implement the multidimensional ranking model in scenarios in which relevance judgments are not available, we proposed multiple (pretrained) models for each of the quality dimensions using transfer learning.

##### Usefulness

In this reranking step, we created an ensemble of pretrained language models—BERT-base, mono-BERT-large, and ELECTRA—all fine-tuned in the MS MARCO [47] data set. Each model then predicted the similarity between the topic and the initial list of retrieved documents. Their results were finally combined using reciprocal rank fusion (RRF) [32].

##### Supportiveness

In this reranking step (Figure 1B), we created an ensemble of claim-checking models—robustly optimized BERT approach (RoBERTa)–Large [48], BioMedRoBERTa-base [49], and SciBERT-base [50]—which were fine-tuned on the FEVER [51] and SciFact [52] data sets. Claim-checking models take a claim and a document as the information source and validate the veracity of the claim based on the document content [53]. Most claim-checking models assume that document content is ground truth. Since this is not valid in the case of web documents, we added a further classification step that evaluates the correctness of the retrieved documents. We used the top-*k* assignments [44] provided by the claim-checking models to define whether the topic should be supported or refuted. The underlying assumption is that a scientific fact is defined by the largest number of evidence available for a topic. A higher rank is then given to the correct supportive or dissuasive documents, a medium rank is given to the neutral documents, and a lower rank is given to the incorrect supportive or dissuasive documents. The rank lists obtained for each model were then combined using RRF.

##### Credibility

In this step, we implemented a random forest classifier trained on the Microsoft Credibility data set [54] with a set of credibility-related features, such as readability, openpage rank [45], and the number of cascading style sheets (CSS). The data set manually rated 1000 web pages with credibility scores between 1 (“very noncredible”) and 5 (“very credible”). We converted these scores for a binary classification setting—that is, scores of 4 and 5 were considered as 1 or *credible*, and scores of 1, 2, and 3 were considered as 0 or *noncredible*. For the readability score, we relied on the Simple Measure of Gobbledygook index [44], which estimates the years of education an average person needs to understand a piece of writing. Following Schwarz and Morris [54], we retrieved a web page’s PageRank and used it as a feature to train the classifier. We further used the number of CSS style definitions to estimate the effort for the design of a web page [55]. Last, a list of credible websites scrapped from the Health On the Net search engine [46] for the evaluated topics was combined with the baseline model to explore better performance. The result of the classifier was added to the unitary value of the Health On the Net credible sites [46].

#### Domain Adaptation Implementation

To implement the multidimensional ranking model in scenarios in which relevance judgments are available, we compared different pretrained language models—BERT, BioBERT [56], and BigBird [57]—for each of the quality dimensions using domain adaptation. In this case, each model was fine-tuned to predict the relevance judgment of a specific dimension (ie, usefulness, supportiveness, and credibility). Although the input size was limited to 512 tokens for the first 2 models, BigBird allows up to 4096 tokens.

We used the TREC 2019 Decision Track [33] benchmark data set to fine-tune our specific quality dimension models. The TREC 2019 Decision Track benchmark data set contains 51 topics evaluated across 3 dimensions: relevance, effectiveness, and credibility. Adhering to the experimental design set by [58], we mapped the 2019 and 2021 benchmarks as follows. The relevance dimension (2019) was mapped to usefulness (2021), with highly relevant documents translated as very useful and relevant documents as useful. The effectiveness dimension (2019) was mapped to supportiveness (2021), with effective labels reinterpreted as supportive and ineffective as dissuasive. The credibility dimension (2019) was directly mapped to credibility (2021) using the same labels.

The 2019 track uses the ClueWeb12-B13 [59,60] corpus, which contains 50 million pages. More details on the TREC 2019 Decision Track [33] benchmark are provided in Table S2 in Multimedia Appendix 1.

In the training phase, the language models received as input were the pair topic-document and a label for each dimension according to the 2019-2021 mapping strategy. At the inference time, given a topic-document pair from the TREC Health Misinformation Track 2021 benchmark, the model would infer its usefulness, supportiveness, or credibility based on the dimension on which it was trained.

#### Preprocessing or Ranking Phase

In the preprocessing step, which is initially executed to select a short list of candidate documents for the input query, a BM25 model was used. This step was performed using a bag-of-words model due to its efficiency. For the C4 snapshot collection, 2 indices were created, one using standard BM25 parameters and another fine-tuned using a collection of topics automatically generated (silver standard) from a set of 4985 indexed documents. For a given document, the silver topic was created based on the keyword2query [61] and doc2query [41] models to provide the query and description content, respectively. Using the silver topics and their respective documents, the BM25 parameters of the second index were then fine-tuned using grid search in a known-item search approach [62] (ie, for a given silver topic, the model should return in the top-1 the respective document used to generate it). The results of these 2 indices were fused using RRF.

### Evaluation Metric

We followed the official TREC evaluation strategy and used the compatibility metric [46] to assess the performance of our models. Contrary to the classic information retrieval tasks, in which the performance metric relies on the degree of relatedness between queries and documents, in quality retrieval, harmful documents should be penalized, especially if they are relevant to the query content. In this context, the compatibility metric calculates the similarity between the actual ranking *R* provided by a model and an ideal ranking *I* as provided by the query relevance annotations. According to Equation 1, the compatibility is calculated with the rank-biased overlap (RBO) [63] similarity metric, which is top-weighted, with greater weight placed at higher ranks to address the indeterminate and incomplete nature of web search results [64]:



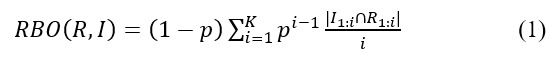



where the parameter *p* represents the searcher's patience or persistence and is set to 0.95 in our experiments and K is the search depth and is set to 1000 to bring *pK*-1 as close to 0 as possible. As shown in Equation 2, an additional normalization step was added to accommodate short, truncated ideal results, so when there are fewer documents in the ideal ranking than in the actual ranking list, it does not influence the compatibility computation results:



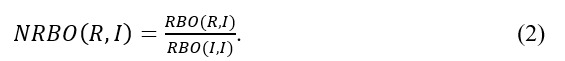



To ensure that helpful and harmful documents are treated differently, even if both might be relevant to the query content, the assessments were divided into “help compatibility” (help) and “harm compatibility” (harm) metrics. To evaluate the ability of the system to separate helpful from harmful information, the “harm compatibility” results were then subtracted from the “help compatibility” results, which were marked as “help-harm compatibility” (help-harm). Overall, the more a ranking is compatible with the ideal helpful ranking, the better it is. Conversely, the more a ranking is compatible with the ideal harmful ranking, the worse it is.

### Experimental Setup

The BM25 indices were created using the Elasticsearch framework (version 8.6.0). The number of documents *N_D_* retrieved per topic in the preprocessing step was set to 10,000 in our experiments. The pretrained language models were based on open-source checkpoints from the HuggingFace platform [65] and were implemented using the open-source PyTorch framework. The language models used for the usefulness dimension and their respective HuggingFace implemations were BERT base (Capreolus/bert-base-msmarco), BERT large (castorini/monobert-large-msmarco-finetune-only), and ELECTRA (Capreolus/electra-base-msmarco). The language models used for the supportiveness dimension were RoBERTa base (allenai/biomed_roberta_base), RoBERTa large (roberta-large), and SciBERT (allenai/scibert_scivocab_uncased). For the credibility dimension, we used the random forest algorithm of the scikit-learn library. In the domain adaptation setup, we partitioned the 2019 labeled data set into training and validation sets using an 80%:20% split ratio; the latter was used to select the best models. We then fine-tuned BioBERT (dmis-lab/biobert-base-cased-v1.1) with a batch size of 16, learning rate of 1^-5^, and 20 epochs with early stopping set at 5 and utilizing the binary cross-entropy loss, which was optimized using the Adam optimizer. The BigBird model (google/bigbird-roberta-base) was fine-tuned with a batch size of 2, keeping all the other settings the same as the BioBERT model. All language models were fine-tuned using a single NVIDIA Tesla V100 graphics card with 32 GB of memory (see Multimedia Appendix 2 for more details). Results are reported using the compatibility and normalized discounted cumulative gain (nDCG) metrics. For reference, they were compared with the results of other participants of the official TREC Health Misinformation 2021 track, which have submitted runs for the automatic evaluation (ie, without using information about the topic stance). The code repository is available at [66].

### Ethical Considerations

No human participants were involved in this research. All data used to build and evaluate the deep language models were publicly available and open aceess.

## Results

### Performance Results

In Table 3, we present the performance results of our quality-based retrieval models using the TREC Health Misinformation 2021 benchmark. Helpful compatibility (help) considers only helpful documents of the relevant judgment, while harmful compatibility (harm) considers only harmful documents and help-harm considers their compatibility difference (see Table S1 in Multmedia Appendix 1 for further detail). Additionally, we show the nDCG scores calculated using helpful (help) documents or harmful (harm) documents of the relevant judgment. The helpful*_T_*, unhelpful*_T_*, and all*_T_* terms denote helpful topics, unhelpful topics, and all topics, respectively. *H_U_*, *H_S_*, and *H_C_* rankings represent the combination of the preprocessing (*H_P_*) results with the rerankings results for usefulness (*H_U_’*), supportiveness (*H_S_’*), and credibility (*H_C_’*), respectively. For reference, we show our results compared with the models participating in the TREC Health Misinformation Track 2021: Pradeep et al [31] used the default BM25 ranker from Pyserini. Their reranking process incorporated a mix of mono and duo T5 models as well as Vera [67] on different topic fields. Abualsaud et al [68] created filtered collections that focus on filtering out nonmedical and unreliable documents, which were then used for retrieval with Anserini’s BM25. Schlicht et al [69] also used Pyserini’s BM25 ranker and Bio Sentence BERT to estimate usefulness and RoBERTa for credibility. The final score was a fusion of these individual rankings. Fernández-Pichel et al [70] used BM25 and RoBERTa for reranking and similarity assessment of the top 100 documents, trained an additional reliability classifier, and merged scores using CombSUM [71] or Borda Count. Bondarenko et al [72] used Anserini’s BM25 and PyGaggle’s MonoT5 for 2 baseline rankings, then reranked the top 20 from each using 3 argumentative axioms on seemingly argumentative queries.

**Table 3:** Performance results for the quality-based retrieval models.

Model			nDCG^a^					Compatibility								
			Help^b^ ↑		Harm^c^ ↓		Help ↑			Harm ↓		Help-harm ↑				
			all_*T*_^d^		all_*T*_		all_*T*_			all_*T*_		helpful_*T*_^e^		unhelpful_*T*_^f^		all_𝑇_
BM25^g^ [39]			0.516		0.360		0.122			0.144		0.158		–0.162		–0.022
Pradeep et al [31]			0.602		0.378		0.195^h^			0.153		0.234^h^		–0.106		0.043
Abualsaud et al [68]			0.302		0.185^h^		0.164			0.123		0.179		–0.067		0.040
Schlicht et al [69]			0.438		0.309		0.121			0.103		0.157		–0.089		0.018
Fernández-Pichel et al [70]			0.603^h^		0.363		0.163			0.155		0.163		–0.113		0.008
Bondarenko et al [72]			0.266		0.226		0.129			0.144		0.150		–0.144		–0.015
**Transfer learning**																
	*H_U_^i^*	0.538^j^		0.324		0.142^j^			0.087^h^		0.156		–0.022^h^		0.056^h^	
	*H*_*U*_ + *H*_*S*_^k^	0.477		0.315^j^		0.130			0.092		0.151		–0.049		0.038	
	*H*_*U*_ + *H*_*S*_ + *H*_*C*_^l^	0.484		0.320		0.137			0.095		0.169^j^		–0.057		0.042	
**Domain adaptation**																
	*H*_*U*_	0.510		0.327		0.128			0.100		0.146		–0.063		0.029	
	*H*_*U*_ + *H*_*S*_	0.482		0.319		0.108			0.089		0.108		–0.050		0.019	
	*H*_*U*_ + *H*_*S*_ + *H*_*C*_^l^	0.502		0.325		0.131			0.094		0.147		–0.048		0.037	

^a^nDCG: normalized discounted cumulative gain.

^b^Help: results considering only helpful documents in the relevance judgment.

^c^Harm: results considering only harmful documents in the relevance judgment.

^d^all*_T_*: all topics.

^e^helpful*_T_*: helpful topics.

^f^unhelpful*_T_*: unhelpful topics.

^g^BM25: Best Match 25.

^h^Best performance.

^i^*H_U_*: usefulness model.

^j^Best performance among our models.

^k^*H_S_*: supportiveness model.

^l^*H_C_*: credibility model.

Our approach provides state-of-the-art results for automatic ranking systems in the transfer learning setting, with help-harm compatibility of +5.6%. This result was obtained with the usefulness model (*H_U_*), which is the combination of preprocessing and usefulness reranking. It outperformed the default BM25 model [39] by 7% (*P*=.04) and the best automatic model from the TREC 2021 benchmark (Pradeep et al [31]) by 1%. In this case, although the help and harm compatibility metrics individually exhibited statistical significance (*P*=.02 and *P*=.01, respectively), the improvement in help-harm compatibility compared with the best automatic model was not statistically significant (*P*=.70). The usefulness model also stood out by achieving the best help and harm compatibility metrics among our models (14.2% and 8.7%, respectively; *P*=.50). Notice that, for the latter metric, the closest to 0, the better the performance. Interestingly, the usefulness model attained the highest nDCG score on help for all topics as well (*P*=.03). The combination of usefulness, supportiveness, and credibility models (*H_U_* + *H_S_* + *H_C_*) provided the best help-harm (+16.9%) for helpful topics among our models (*H_U_*: *P*=.40; *H_U_* + *H_S_*: *P*=.04).

Meanwhile, when calculating nDCG scores on harm, the combination of usefulness and supportiveness model (*H_U_* + *H_S_*) in the transfer learning and domain adaption settings outperformed the other model combinations (*P*=.50), indicating a different perspective of the best-performing model. Last, differently from what would be expected, in the domain adaption setting, the performance was poorer than the simpler transfer learning approach (2% decrease on average for the compatibility metric; *P*=.02). See Table S4 in Multimedia Appendix 3 for more information about using nDCG as a metric in a multidimensional evaluation.

### Performance Stratification by Quality Dimension

In Table 4, we show the help, harm, and help-harm compatibility scores for the individual quality-based reranking models, which disregarded the preprocessing step (prime index). Additionally, we provide the nDCG scores for a more comprehensive view of the models’ performance. *H_P_* represents the preprocessing, and *H_U_’*, *H_S_’*, and *H_C_’* stand for rerankings for usefulness, supportiveness, and credibility, respectively.

**Table 4:** Performance results for the individual ranking models.

Setting and model			nDCG^a^					Compatibility								
			Help^b^ ↑		Harm^c^ ↓		Help ↑			Harm ↓		Help-harm ↑				
			all_*T*_^d^		all_*T*_		all_*T*_			all_*T*_		helpful_*T*_^e^		unhelpful_*T*_^f^		all_*T*_
*H_P_^g^*			0.538^h^		0.341		0.126^h^			0.111		0.127^h^		–0.072		0.015
**Transfer learning**																
	*H_U_’^i,j^*	0.438		0.264		0.115			0.080		0.106		–0.020		0.036	
	*H_S_’^j,k^*	0.140		0.102^h^		0.026			0.024		0.021		–0.013		0.002	
	*H_C_’^j,l^*	0.131		0.113		0.031			0.035		0.033		–0.032		–0.003	
**Domain adaptation**																
	*H_U_’*	0.436		0.277		0.077			0.038		0.099		–0.008		0.039^h^	
	*H_S_’*	0.368		0.251		0.030			0.015^h^		0.030		0.003^h^		0.014	
	*H_C_’*	0.443		0.296		0.079			0.064		0.104		–0.055		0.014	

^a^nDCG: normalized discounted cumulative gain.

^b^Help: results considering only helpful documents in the relevance judgment.

^c^Harm: results considering only harmful documents in the relevance judgment.

^d^all*_T_*: all topics.

^e^helpful*_T_*: helpful topics.

^f^unhelpful*_T_*: unhelpful topics.

^g^*H_p_*: preprocess.

^h^Best performance.

^i^*H_U_’*: usefulness model.

^j^Unlike *H_U_*, *H_S_*, and *H_C_*_,_*H_U_*’, *H_S_’*, and *H_C_’* rankings are not combined with *H_p_.*

^k^*H_S_’*: supportiveness model.

^l^*H_C_’*: credibility model.

In the transfer learning setting, the usefulness model (*H_U_’*) achieved the highest help-harm compatibility (+3.6%; *P*=.20). The preprocessing model gave the best help compatibility (+12.7%; *H_U_’*: *P*=.70; *H_S_’* and *H_C_’*: *P*<.001). Additionally, the preprocessing model yielded the highest nDCG score for help (*H_U_’*: *P*=.10; *H_S_’* and *H_C_’*: *P*<.001). On the other hand, the preprocessing model showed the highest harm compatibility (+11.1%; *H_U_’*: *P*=.33; *H_S_’* and *H_C_’*: *P*<.01). The combination of the preprocessing and usefulness models (ie, *H_U_*=+5.6%) improved the preprocessing model by 4.1% (from +1.5% to +5.6% on the help-harm compatibility; *P*=.06). For harm compatibility, the supportiveness model (*H_S_’*) achieved the best performance among the individual models (+2.4%; *Hp*: *P*<.001; *Hu’*: *P*=.03; *H_C_’*: *P*=.34)*.*

In the domain adaptation setting, the usefulness model (*H_U_’*) reached help-harm compatibility of +3.9%, similarly outperforming the other models (*P*=.32). The supportiveness model (*H_S_’*) achieved the best performance on harm compatibility (+1.5%; *P*=.07) and on help-harm compatibility for unhelpful topics (+0.3%; *P*=.50). Notice that +0.3% is the only positive help-harm compatibility for harmful topics throughout all the individual and combined models on both settings including the preprocessing step. Last, in the domain adaption setting, the performance of individual models was better than the simpler transfer learning approach (1% increase on average for the compatibility metric; *P*=.19).

### Reranking of the Top-N Documents

**Figure 2:**
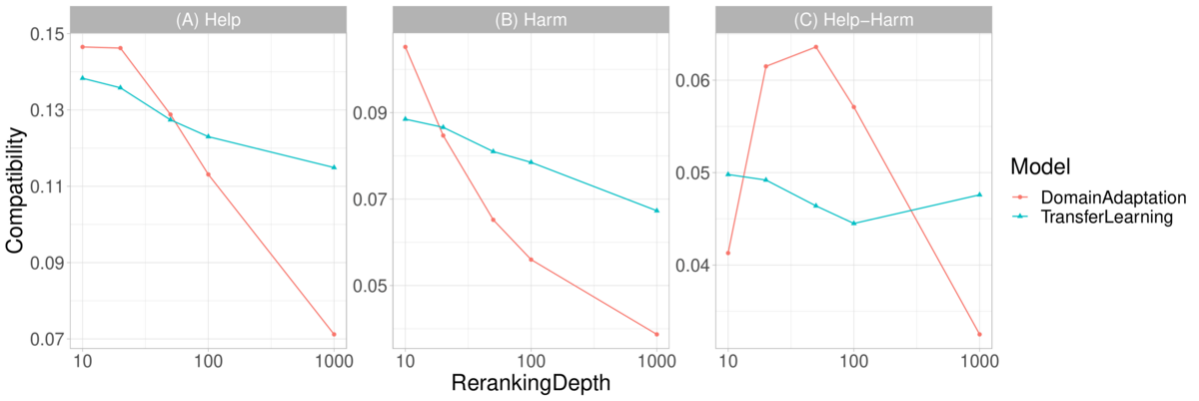
Compatibility performance for the top 10, 20, 50, 100, and 1000 reranking depths taking the results of usefulness as the basis.

To further illustrate the effectiveness of the supportiveness and credibility dimensions, in Figure 2, we reranked only the top-n documents using the results of the usefulness model (*H_U_*) as the basis. As we can see in Table 4, the overall effectiveness of the supportiveness (*H_S_’*) and credibility (*H_C_’*) models were considerably lower than that of the usefulness (*H_U_’*) model. The reason is that the relevance judgments were created using a hierarchical approach: Only useful documents were further considered for supportiveness and credibility evaluations. As we reranked the documents in supportiveness and credibility dimensions without taking this hierarchy into account, their results might not be optimal. For example, low-ranking documents (ie, not useful) could have high credibility and, during the reranking process, could be boosted to the top ranks. Thus, we applied the supportiveness (*H_S_’*) and credibility (*H_C_’*) models to the usefulness model (*H_U_*) results to rerank the top 10, 20, 50, 100, and 1000 documents, obtaining 2 new rankings, which were combined using RRF.

As the reranking depth increased from 10 to 1000, we observed a decrease in both help and harm compatibility. This suggests that both helpful and harmful documents were downgraded due to the inclusion of less useful but potentially supportive or credible documents. In the transfer learning setting, as the reranking depth increased, the help-harm compatibility decreased until the depth reached 100. Beyond this point, we observed a slight increase at the depth of 1000. In the domain adaptation setting, the help-harm compatibility increased above +6% when the reranking depth was between 20 and 50. This implies that, following the procedure of human annotation, by considering only the more useful documents, the supportiveness and credibility dimensions can help retrieve more helpful than harmful documents.

### Quality Control

**Figure 3:**
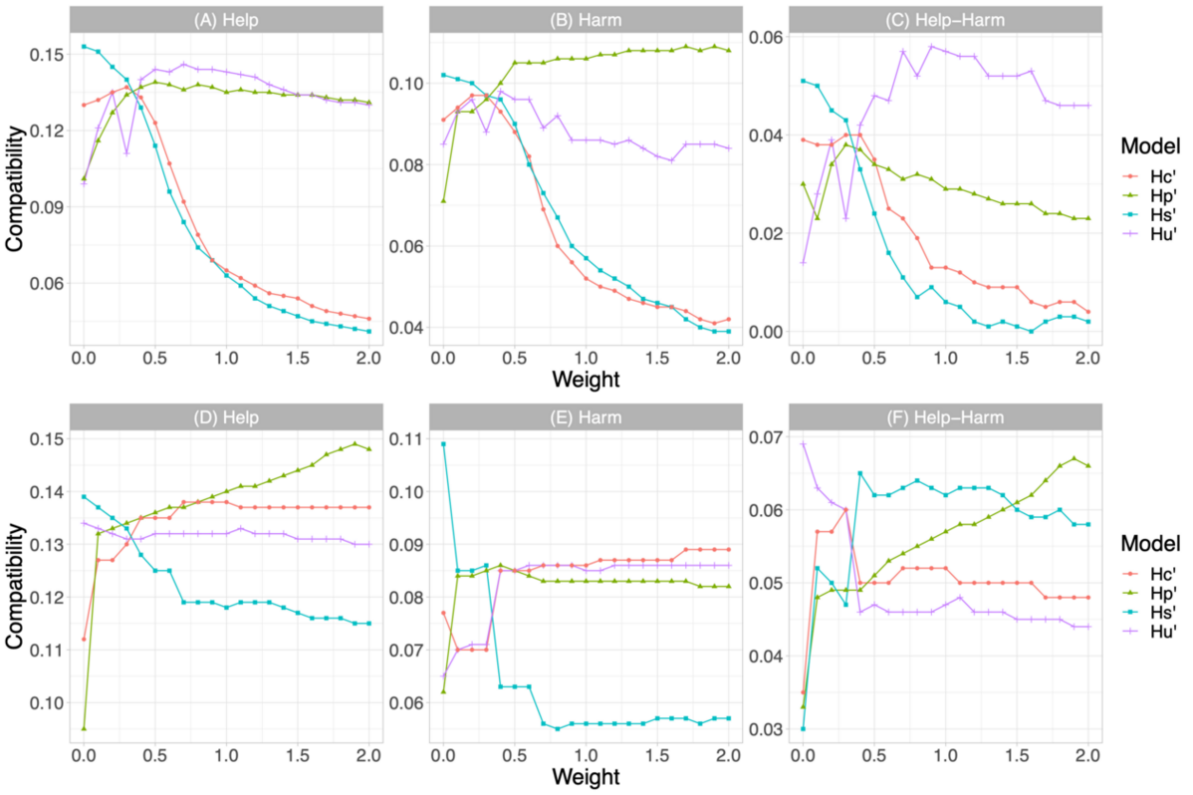
Compatibility in the transfer learning approach (A-C) and compatibility in the domain adaptation approach (D-F), all with weights added to specific models.

One of the advantages of the proposed multidimensional model is that we can optimize the results according to different quality metrics. In Figure 3, we show how the compatibility performance varies by changing the weight of the specific models (*H_P_*, *H_U_’*, *H_S_’*, and *H_C_’*). We normalized the score of the individual models to the unit and combined them linearly using a weight for 1 model between 0 and 2 while fixing the weight for the other 3 models at 0.33. For example, to see the influence of *H_P_* in the final performance, we fixed the weights of *H_U_’*, *H_S_’*, and *H_C_’* at 0.33 and varied the weight of *H_P_* between 0 and 2. With weight 0, the reference model did not account for the final rank, while with weight 2, its impact was twice the sum of the other 3 models.

In the transfer learning setting, when we increased the weight of preprocessing and usefulness models, the help-harm compatibility increased to the best performance (+4.1% and +5.6%) then decreased slightly. For the supportiveness and credibility dimensions, the help-harm compatibility began to decrease once the weight was added. These results imply that the compatibility decreases with the weight addition regardless of whether it is helpful compatibility, harmful compatibility, or the difference between the 2.

In the domain adaptation setting, when we increased the weight of preprocessing, supportiveness, and credibility models individually, the help-harm compatibility increased then converged to +6.6%, +5.9%, and +4.8%, respectively. For the usefulness model, the help-harm compatibility decreased once the weight was added until it converged to +4.4%. It is worth noticing that, by combining the rankings linearly, the help-harm compatibility obtained from the domain adaptation setting may exceed the results we obtained when performing ranking combination with RRF (+3.7%), as well as the state-of-the-art result (+5.6%) in the transfer learning setting. The highest help-harm compatibility scores for each weighting combination were +6.6%, +6.8%, +6.5%, and +5.9% when varying the weights of *H_P_*, *H_U_’*, *H_S_’*, and *H_C_’*, respectively.

### Model Interpretation

To semantically explain the variation of help-harm compatibility, we set the search depth 𝐾 to 10. The help, harm, and help-harm compatibility of the 3 models are shown in Table 5. The help-harm compatibility was 1 when only helpful documents were retrieved in the top 10. Conversely, the help-harm compatibility was –1 when only harmful documents were retrieved in the top 10. A variation of 10% in the help or harm compatibility corresponded roughly to 1 helpful document exceeding the number of harmful documents retrieved in the top 10. Overall, the results show that retrieving relevant documents for health-related queries is hard, as, on average, only 1.5 of 10 documents were relevant (helpful or harmful) to the topic. In addition, we interpreted that the 3 models retrieved, on average, twice the number of helpful documents as harmful documents. Particularly, *H_U_* had, on average, around 1 more helpful than harmful document in the top 10, of the 1.5 relevant documents retrieved. We also present the same analysis results for the domain adaptation setting, which also implies that, when the rankings were combined with RRF, the transfer learning approach outperformed the domain adaptation approach. See more details about the average compatibility for all the topics as the search depth K varied in Figure S1 in Multimedia Appendix 3.

**Table 5:** Help, harm, and help-harm compatibility with search depth set to 10 for the transfer learning setting and domain adaptation setting.

Setting and model		Help^a^ ↑	Harm^b^ ↓	Help-harm ↑
**Transfer learning**				
	*H_U_^c^*	0.112^d^	0.047^d^	0.065^d^
	*H*_*U*_ + *H*_*S*_^e^	0.088	0.050	0.038
	*H*_*U*_ + *H*_*S*_ + *H*_*C*_^f^	0.099	0.056	0.044
**Domain adaptation**				
	*H*_*U*_	0.094	0.060	0.034
	*H*_*U*_ + *H*_*S*_	0.074	0.070	0.003
	*H*_*U*_ + *H*_*S*_ + *H*_*C*_	0.087	0.076	0.011

^a^Help: results considering only helpful documents in the relevance judgment.

^b^Harm: results considering only harmful documents in the relevance judgment.

^c^*H_U_*: usefulness model.

^d^Best performance.

^e^*H_S_*: supportiveness model.

^f^*H_C_*: credibility model.

## Discussion

We propose a quality-based multidimensional ranking model to enhance the usefulness, supportiveness, and credibility of retrieved web resources for health-related queries. By adapting our approach in a transfer learning setting, we showed state-of-the-art results in the automatic quality ranking evaluation benchmark. We further explored the pipeline in a domain adaptation setting and showed that, in both settings, the proposed method can identify more helpful than harmful documents, as measured by +5% and +7% help-harm compatibility scores, respectively. By combining different reranking strategies, we showed that multidimensional aspects have a significant impact on retrieving high-quality information, particularly for unhelpful topics.

The quality of web documents is biased in terms of topic stance. For all models, helpful topics achieve higher help compatibility, while unhelpful topics achieve higher harm compatibility. The implication is that web documents centered around helpful topics are more likely to support the intervention and are helpful. On the other hand, web documents focusing on unhelpful topics present an equal chance of being supportive or dissuasive on the intervention and are helpful or harmful. Among other consequences, if web data are used to train large language models without meticulously crafted training examples using effective data set search methods [73], as the one proposed here, they are likely to further propagate health misinformation.

Automatic retrieval systems tend to find more helpful information on helpful topics with the information biased toward helpfulness and find more harmful information on unhelpful topics with the information slightly biased toward harmfulness. The help-harm compatibility ranged from +2.3% to +15.3% for helpful topics and from –5.7% to +0.2% for unhelpful topics. The difference shows that, for the improvement of quality-centered retrieval models, it is especially important to focus on unhelpful topics. Moreover, although specialized models might provide enhanced effectiveness, their combination is not straightforward. In our experiments, we showed that supportiveness and credibility models should be applied only in the top 20 to 50 retrieved documents to achieve optimal performance.

Finding the correct stance automatically is another key component of the automatic model. Automatic models show the ability to prioritize helpful documents, resulting in positive help-harm compatibility. However, they are still far from state-of-the-art manual models, with help-harm compatibility scores ranging from +20.8% [68] to +25.9% [31]. We acknowledge that the help-harm compatibility can improve significantly with the correct stance given. This information is nevertheless unavailable in standard search environments; thus, the scenario analyzed in this work is more adapted to real-world applications.

This work has certain limitations. In the domain adaptation setting, we simplified the task to consider 2 classes within each dimension for the classification due to the limited variety available in the labeled data set. Alternatively, we could add other classes from documents that have been retrieved. Moreover, the number of topics used to evaluate our models was limited (n=32), despite including 6030 human-annotated, query-document pairs, and thus reflects only a small portion of misinformation use cases.

To conclude, the proliferation of health misinformation in web resources has led to mistrust and confusion among online health advice seekers. Automatic maintenance of factual discretion in web search results is the need of the hour. We propose a multidimensional information quality ranking model that utilizes usefulness, supportiveness, and credibility to strengthen the factual reliability of health advice search results. Experiments conducted on publicly available data sets show that the proposed model is promising, achieving state-of-the-art performance for automatic ranking in comparison with various baselines implemented on the TREC Health Misinformation 2021 benchmark. Thus, the proposed approach could be used to improve online health searches and provide quality-enhanced information for health information seekers. Future research could explore more granular classification models for each dimension, and a model simplification could provide an advantage for real-world implementations.

## Appendix

Multimedia Appendix 1 Additional Information on Benchmark Datasets.

Multimedia Appendix 2 Fine-Tuning in the Domain Adaptation Setting.

Multimedia Appendix 3 Supporting Experiment Results.

## Abbreviations

BERT: Bidirectional Encoder Representations from Transformers
BM25: Best Match 25
C4: Colossal Clean Crawled Corpus
CLEF: Conference and Labs of the Evaluation Forum
CSS: cascading style sheets
nDCG: normalized discounted cumulative gain
NIST: National Institute of Standards and Technology
RBO: rank-biased overlap
RoBERTa: robustly optimized BERT approach
RRF: reciprocal rank fusion
TREC: Text Retrieval Conference

## Data Availability

The data sets generated during and/or analyzed during this study are available in the Text Retrieval Conference (TREC) Health Misinformation Track repository [74] and GitLab repository [66].
