# Supplementary material for: Online Health Search Via Multidimensional Information Quality Assessment Based on Deep Language Models: Algorithm Development and Validation
Source: JMIR AI. 2024 May 2;3:e42630. doi: 10.2196/42630 (PMC11099810; doi:10.2196/42630)
Supplement: Multimedia Appendix 1 [file ai_v3i1e42630_app1.pdf]

1 Additional Information on Benchmark Datasets

Table S1. Dimensions for assessing topic-Web document pairs and their respective assessment score.

**Correctness**	Correct		Neutral		Incorrect
**Usefulness** / **Credibility**	Very useful	Useful	Very useful	Useful	Very useful, Useful
Excellent	12	11	6	5	-3
Good	10	9	4	3	-2
Low	8	7	2	1	-1
**Document**	Helpful				Harmful

Table S2. Mappings of labeled data for training/validation (2019) and test (2021).

**Dataset**	**Usefulness**		**Supportiveness**		**Credibility**	
	Very Useful	Useful	Support	Refute	Credible	Not Credible
Train (2019)	826	1832	2572	86	1417	1238
Dev (2019)	113	414	452	75	315	212
Test (2021)	2089	2467	3667	889	2831	1706
