# Supplementary material for: Online Health Search Via Multidimensional Information Quality Assessment Based on Deep Language Models: Algorithm Development and Validation
Source: JMIR AI. 2024 May 2;3:e42630. doi: 10.2196/42630 (PMC11099810; doi:10.2196/42630)
Supplement: Multimedia Appendix 2 [file ai_v3i1e42630_app2.pdf]

2 Fine-Tuning in the Domain Adaptation Setting

Statement Generation

We convert the query from a question to a statement format. We tokenize the query using the SpaCy scispacy library (en_core_sci_sm) and extract name entities. Each entity is treated as a single token and the first two tokens are swapped if the initial token is a question word. For instance, the query 'Is a tepid sponge bath a good way to reduce fever in children?' is translated automatically into 'a tepid sponge bath a good way to reduce fever in children.'.

Sentence Selection

Given the maximum token limit at 512 of a BERT model, with the same model as the encoder, we calculate the cosine similarity between the statement and sentence representations. We rank sentences by their relevance to the statement and extract the most significant text compiled until reaching the token limit of 512. For BioBERT, we implement two methods: truncating retrieved documents to 512 tokens (TD) and using sentence selection (SS).

Ranking Strategies

Table S3: Classification accuracy and F1 score on the validation set. (TD: truncating retrieved documents; SS: sentence selection; Note: Data in this table is compared row-wise.)

Metrics	Dimension	BioBERT (TD)	BioBERT (SS)	BigBird
Accuracy	Usefulness	0.776	**0.801**	0.685
	Supportiveness	0.800	**0.880**	0.858
	Credibility	0.490	0.615	**0.681**
F1-macro	Usefulness	0.540	0.331	**0.605**
	Supportiveness	0.601	**0.932**	0.462
	Credibility	0.489	0.565	**0.628**

Based on the F1 Marco scores in Table S3, we select the best-performing model for each dimension to re-rank the preprocessing ranking list. For usefulness and credibility, the positive confidence score is used when the predicted label is very useful or credible, while the negative score is used when the predicted label is useful or not credible. For supportiveness, we utilize the top-10 most credible documents to determine the stance of the topic. If the stance of the topic matches the stance

of the document, we use the positive confidence score, otherwise we use the negative confidence score. We employ these updated confidence scores to re-rank the documents for each dimension.
