# Supplementary material for: Online Health Search Via Multidimensional Information Quality Assessment Based on Deep Language Models: Algorithm Development and Validation
Source: JMIR AI. 2024 May 2;3:e42630. doi: 10.2196/42630 (PMC11099810; doi:10.2196/42630)

3 Supporting Experiment Results

Table S4: nDCG usefulness, correctness, and credibility across quality-based retrieval models.

**Setting**	**Model**	**nDCG**		
		Useful	Correct	Credible
Transfer Learning	H_U	0.595	**0.596**	0.514
	$H_U + H_S$	0.559	0.546	0.468
	$H_U + H_S + H_C$	0.564	0.557	0.486
Domain Adaptation	H_U	**0.607**	0.566	0.547
	$H_U + H_S$	0.578	0.540	0.529
	$H_U + H_S + H_C$	0.58**6**	0.563	**0.563**

We use nDCG as a metric to provide another perspective on evaluating usefulness, correctness, and credibility. The usefulness model (H_U) in the transfer learning setting shows the highest nDCG for correctness (0.596), which implies that correctness can already be leveraged by improving only the usefulness dimension of the model. In the domain adaptation setting, the usefulness model (H_U) demonstrates the highest nDCG for usefulness (0.607). The combination of usefulness, supportiveness, and credibility models ($H_U + H_S + H_C$) presents the highest nDCG for credibility (0.563). The results imply that the usefulness and credibility dimensions each effectively contribute to their respective attributes in the retrieved documents.

In Figure S1, we show the average compatibility for all the topics as the search depth K varies. In the transfer learning setting, when the search depth is 1, we compare the top-1 document in the ranking list and the query relevance set. In this case, the compatibility is either 0 or 1 for each topic. When the search depth is above 1, we compare the top-K documents and the compatibility for each topic varies between 0 and 1. The compatibilities increase almost monotonically until the search depth K becomes 100 as the query reaches the average number of documents in the relevance set (i.e., 92 documents) and subsequently becomes saturated. Interestingly, we observe that for help-harm compatibility, the model H_U spikes at the depth of 3 due to the higher proportion of helpful documents retrieved as compared to harmful documents. Further, with the depth increase, we notice

that the difference in proportions of helpful and harmful documents diminishes steeply between depths 3 to 10. The results in the domain adaptation setting indicate the same trend.

Figure S1. Help, harm, and help-harm compatibility with different search depths. a) Transfer learning approach. b) Domain adaptation approach.

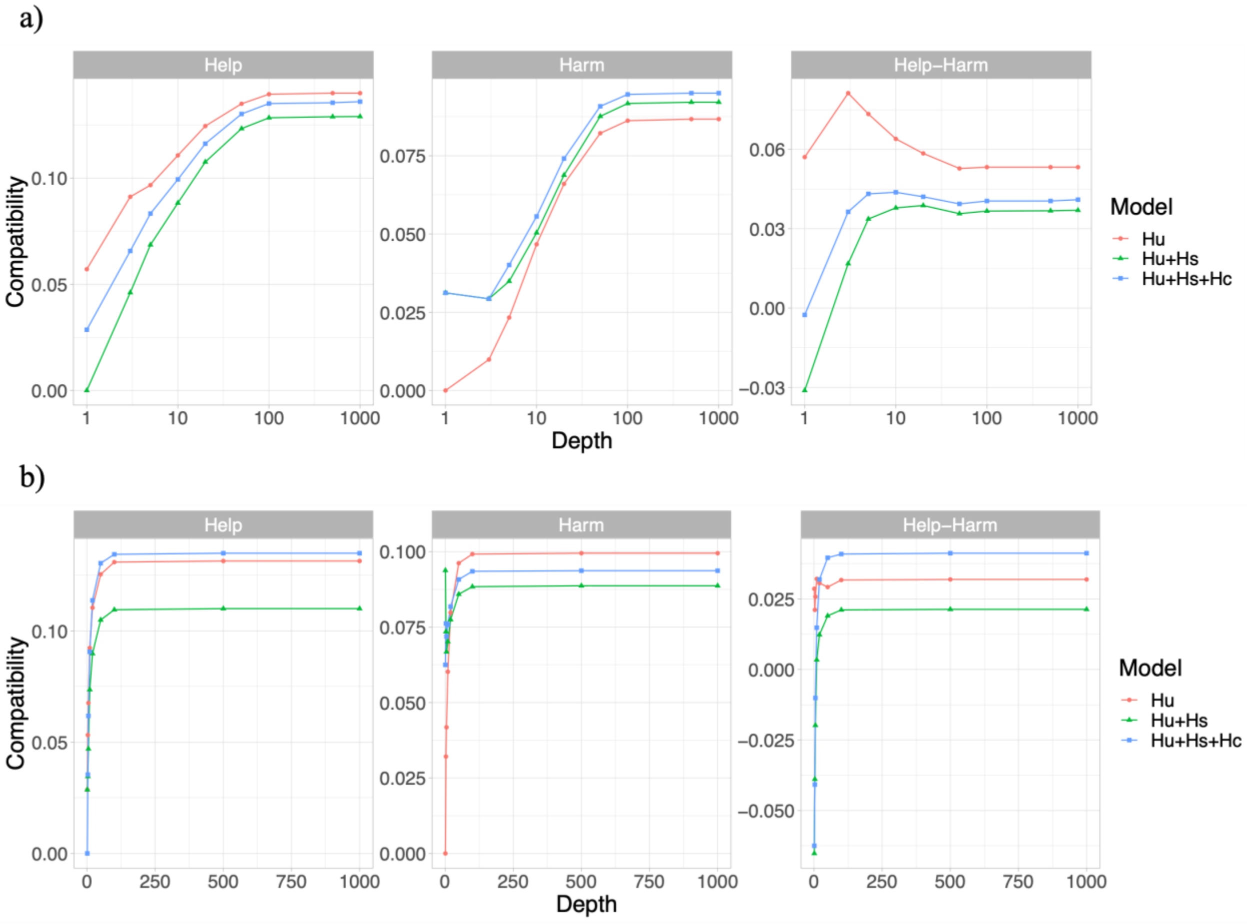

Supplement: Multimedia Appendix 3 [file ai_v3i1e42630_app3.pdf]
